# Knowledge, attitude and practice on fall risk factors and prevention among rural older community-dwellers in Vietnam

**DOI:** 10.1371/journal.pone.0295119

**Published:** 2023-11-30

**Authors:** Hao Thi Tang, Hai Minh Vu, Hai Thi Tang, Phuc Thai Tran, Long Van Tran, Cuong Duy Nguyen, Tien Quoc Nguyen, Chinh Minh Thi Nguyen, Kham Quoc Tran, Hien Xuan Luong

**Affiliations:** 1 Faculty of Nursing, Nam Dinh University of Nursing, Nam Dinh, Vietnam; 2 Nursing Department, Thai Binh University of Medicine and Pharmacy, Thai Binh, Vietnam; 3 Department of Trauma, Thai Binh University of Medicine and Pharmacy, Thai Binh, Vietnam; 4 Physiology Department, Thai Binh University of Medicine and Pharmacy, Thai Binh, Vietnam; 5 Organization and Administration Department, Nam Dinh University of Nursing, Nam Dinh, Vietnam; 6 Department of Intensive care Unit, Thai Binh University of Medicine and Pharmacy, Thai Binh, Vietnam; 7 Faculty of Public Health, Thai Binh University of Medicine and Pharmacy, Thai Binh, Vietnam; 8 Simulation Center, Nam Dinh University of Nursing, NamDinh, Vietnam; Tokyo Medical and Dental University: Tokyo Ika Shika Daigaku, JAPAN

## Abstract

Falls among the elderly are an important global health problem. This study assesses knowledge regarding risk factors of falls, as well as attitudes and practices towards fall prevention among older adults in the rural community. A cross-sectional study was performed in four rural communes in Thai Binh province, Vietnam. A total of 3038 older people were recruited. Knowledge was assessed by using Falls Risk Awareness Questionnaire. Questions about attitudes were based on the Health Belief Model. Other questions regarding attitudes and practices regarding fall prevention were also asked. Multivariate regression was performed to identify associated factors with knowledge, attitudes and practices. Results showed that the mean score of knowledge regarding risk factors of falls was low at 11.37/32. The highest scores were observed in terms of drug aspects, followed by medical condition and behavioural aspects. Older people mostly agreed with perceived severity, susceptibility, benefits and barriers, but their attitudes about cues to action, health motivation and actions were most neutral. Meanwhile, more than half of the participants practised recommended fall preventive measures. To conclude, health education interventions and fall prevention services that enhance community-based fall prevention knowledge, attitudes and practices for older adults should be performed to reduce the burden of falls in this population.

## Introduction

Falls among older people hold considerable weight as a substantial health concern, having made substantial contributions to both older individuals’ injury rates and mortality rates [1]. Falls indicate the primary factor contributing to both morbidity and mortality in individuals aged 60 years and older [1]. The global annual mortality rate due to falls in the elderly population is estimated to be 684,000 individuals, with developing countries accounting for over 80% of these fatalities [1]. The occurrence of falls in the elderly population exhibits an upward trend in correlation with advancing age. Approximately 28–35% of individuals aged 65 years and above experience at least one fall annually, whereas this percentage escalates to 32–42% for individuals above the age of 70, particularly those belonging to the over-85 age demographic [2]. A notable proportion of falls, constituting around 5–10%, culminate in head injuries or fractures, thereby constituting the primary reason behind hospitalization among older adults [3, 4]. In addition to physical injury, falls engender detrimental health consequences, impede independent functioning, augment the burden placed upon caregivers, and impinge upon overall quality of life. The economic ramifications stemming from falls escalated from $50 billion in 2015 to $67.7 billion in 2020 [5, 6].

The comprehension, perspectives, and behaviours regarding fall prevention among older individuals significantly contribute to the identification of fall hazards and proactive engagement in fall prevention endeavours. However, numerous studies have demonstrated the continued existence of knowledge, attitude and practice disparities in fall prevention among the elderly, even in the developed nations. For example, a study in the United States indicated that a significant proportion of the participants, approximately one-third, considered falling to be of minimal importance to their overall health concerns. Furthermore, the majority of individuals possessed limited familiarity with established falling prevention practices that have been scientifically proven [7]. Another study in Ottawa showed that older people exhibited a limited level of knowledge on the potential benefits of undergoing an annual medication review, annual eye and physical examination, as well as daily vitamin D supplementation, in mitigating the risk of falls [8]. To reduce the occurrence of falls among elderly individuals, the crucial aspect involves equipping them with knowledge and skills that enable self-monitoring, identification of risk factors, and proactive prevention of falls [4, 9, 10]. The World Health Organization (WHO) has classified this as one of the three fundamental components within the fall prevention framework for older individuals [2].

In Vietnam, there is projected to be a significant increase in the proportion of individuals aged 60 and above, comprising approximately 25% of the overall population by the year 2049 [11]. In community, the rate of falls among older people were 15% [12]. Meanwhile, the rate of outpatients aged 60 years and older experiencing falls was 23.1% to 23.7% of [13, 14], and 40.5% had recurrent falls within 12 months [15]. Various factors have been identified as having an association with falls among the elderly population. These include, but are not limited to, being underweight, experiencing limitations in instrumental activities of daily living, struggling with poor sleep quality, and harboring a fear of falling. Furthermore, the absence of having received fall prevention guidelines, dependence on walking aids, the presence of sensory impairments in the hands or feet, and the utilization of pain relief medications have all been found to contribute to an increased risk of falling [12–14]. The aforementioned trend poses a significant burden on the healthcare system unless proactive measures are devised to reduce falls among the elderly population. However, currently in Vietnam, there is a lack of specific strategies for preventing falls in the elderly population. The National Strategy for Elderly Health Care by the Government of Vietnam until 2030 solely focuses on enhancing the self-care capabilities of the elderly population [16]. The limited evidence on falls, as well as the knowledge, attitudes, and practices related to fall risk factors and fall prevention in Vietnam contribute to this situation. This study assesses knowledge regarding risk factors of falls, as well as attitudes and practices towards fall prevention among older adults in the rural community. The results of this study would provide some recommendations and directions for future interventions.

## Materials and methods

### Study settings and participants

From January to December 2022, a cross-sectional study was conducted within four communes in Vu Thu district, Thai Binh province. According to Vietnam’s law, older people are defined as people who are aged 60 years old or above. Therefore, participants were eligible for this study if they were aged 60 years or older, lived in the study area for at least 12 months, were able to understand and respond to the questionnaire by themselves, were able to walk and voluntarily participated in the study. Exclusion criteria included people who were unable to walk, were absent at the time of the study and disagreed to participate in the study. A multi-stage sampling method was performed. First, we had a list of all rural communes in the Vu Thu district. Then, four communes were randomly selected for the study comprising: Tan Hoa, Song Lang, Tu Tan, and Bach Thuan communes. More than 8,000 people were living in each commune, with about 800–1000 older people per commune. Based on the support of health workers in each district health centre, we selected all eligible older people in four communes. A formula to estimate a population proportion was used for calculating the sample size. With p = 0.37 (proportion of older people having correct practices of safety management in a previous study [7]), α = 0.05 and ɛ (relative precision) = 0.05, the necessary sample size was 2617 older people. We added 20% more for preventing dropout during invitation and interview processes, resulting in a total of 3140 older people who were invited to participate in the study. Finally, 3038 older people visited the commune health stations to participate in the study (response rate 96.8%).

### Data collection

The data collection team included researchers and medical students of the Thai Binh University of Medicine and Pharmacy, as well as health workers in four commune health stations. All data collectors received intensive training sessions under field experts’ guidance for mastering the interview and communication skills. They were introduced to the study purposes and characteristics of the questionnaire. Supervisors included the principal investigators and the head of the commune health station. The supervisors participated in a training class with the investigators to master the content and supervising methods. The pilot study was performed with ten older people living in the study area. Data collection was performed at the local commune health stations. Older people were invited to the stations by the local health workers. After having the health check-up, participants were invited to a private room for the interview. They were briefly introduced to the study and asked to sign the informed consent forms. Each interview lasted 30 to 40 minutes.

A structured questionnaire was utilized for data collection. The questionnaire was piloted and culturally validated regarding text, logical order of items and languages based on the feedback of local health workers and older people. The questionnaire was then modified, and the final questionnaire was approved by the principal investigators and heads of commune health stations. The questionnaire included five sections: demographic characteristics, fall risk assessment, knowledge of risk factors of falls, attitude towards fall prevention and fall prevention practices.

### Demographic and behavioural information

This section included information about age, gender (male/female), occupation(Retired/Employed or Self-employed), education level (illiteracy/Below primary education/Primary education/Secondary education/High school education/Vocational training or higher), marital status (Single/Have a spouse/Separate/divorce/Widow), Living arrangement (living in 3-/2-/1-generation family, living alone or others), history of falling in the last 12 months, and using assistive tools utilization (including cane, crutch, mobility support framework or others). Participants were also asked to self-report their alcohol use, tobacco smoking, and physical exercise.

### Fall risk assessment

In this study, a Centers for Disease Control and Prevention (CDC)-2017 instrument with 12 items was used for evaluating the risk of falls among participants. Each item had two options for response including “Yes” (= 1 point) and “No”. (= 0 points). Participants had the risk of falling if they had a total score of 4 points or more [17, 18].

### Knowledge of risk factors of falls

The Falls Risk Awareness Questionnaire (FRAQ) was used to assess participants’ knowledge about risk factors related to falls. A total of 22 items were asked in four aspects: medical condition (6 items), behavioural (8 items), drug (3 items), and environmental (5 items) aspects. The score ranged from 0–32 points, and a higher score meant better knowledge regarding risk factors of fall [19].

### Attitudes towards fall prevention

Twenty-six items were employed to assess attitudes towards fall prevention according to the seven components of Health Belief Model (HBM): 1) Perceived Severity (4 items); 2) Perceived Susceptibility (5 items); 3) Perceived Benefits (3 items); 4) Perceived Barriers (5 items); 5) Cues to Action (5 items); 6) Health motivation (2 items) and 7) Action (4 items). Items in six components except Perceived Barriers were responded to on a 5-level Likert scale (1 = Totally disagree; 2 = disagree; 3 = neutral; 4 = agree; and 5 = Totally agree), while responses of items in the Perceived Barriers were reversed (1 = Totally agree, 2 = agree, 3 = neutral, 4 = disagree and 5 = Totally disagree). The score of each component was computed by summing the score of each item in the component. The total attitude score was computed by summing the scores of all components. A higher score indicated a better attitude.

### Practices on fall prevention

Fourteen items were used regarding participants’ practices for preventing falls. Each item had two options for answer including “Yes” (= 1 point) and “No” (= 0 point). The possible score ranged from 0 to 14, with a higher score indicating better practices.

### Statistical analysis

Data were cleaned and entered using Epidata 3.1 software and analyzed using SPSS 20.0 software. Descriptive statistics were performed with mean and standard deviations for quantitative variables and percentage and frequency for qualitative variables. Multivariate linear regression analysis was used to assess factors related to participants’ knowledge, attitudes, and practices. Dependent variables included scores regarding knowledge of risk factors of fall, attitudes toward fall prevention and fall prevention practices. Independent variables included age, gender, occupation, education, marital status, living arrangement history of fall in the last 12 months, and risk of fall (according to the CDC-2017 instrument). The stepwise forward strategy was applied, with a p-value of likelihood test at 0.2 as a threshold for selecting variables into the model. The p-value < 0.05 was used to assess statistical significance.

### Ethical approval

The study was approved by the People’s Committee of Thai Binh Province (2320/QDUNND) and the Thai Binh University of Medicine and Pharmacy. The participants’ information was confidential and used for research purposes only. All participants were informed of the study and signed a written consent form when participating in the study.

## Results

Out of 3038 participants, the mean age was 72.31 ±8.39. The number of males was fewer than females with a male/female ratio of approximately 1/1.57. People with secondary school education were the majority at 44.4%. Elderly people with spouses accounted for the highest proportion at 82%. Living with a three-generation family accounted for the highest proportion of 50.8%. The older people currently working accounted for 64%. There were 35.3% of the participants experienced falls at least once and 47.8% of the participants were at risk of falling. The proportion of participants with physical exercise habits, smoking and drinking alcohol accounted for 66.1%, 18.3%, and 21.4% respectively (Table 1)

**Table 1:** Characteristics of respondents (n = 3038).

Characteristics	n	%
**Gender**		
Male	1184	38.97
Female	1854	61.03
**Age group (years old)**		
60–69	1355	44.60
70–79	1061	34.92
≥ 80	622	20.47
**Occupation**		
Retired	1094	36.01
Employed or Self-employed	1944	63.99
**Education**		
Illiteracy	33	1.09
Below primary education	257	8.46
Primary education	1041	34.27
Secondary education	1349	44.40
High School education	253	8.33
Vocational training or higher	105	3.46
**Marital status**		
Single	90	2.96
Have a spouse	2491	81.99
Separation/divorce	34	1.12
Widow	423	13.92
**Living arrangement**		
3-generation family	1543	50.79
2-generation family	834	27.45
1-generation family	576	18.96
Living alone	78	2.57
Others	7	0.23
**Have a history of falling**	1071	35.25.
**Fall risk assessment**		
No risk	1586	52.21
At risk	1452	47.79
**Use assistive tools**		
Cane	528	17.38
Crutch	144	4.74
Mobility support framework	108	3.55
Other instruments	67	2.21
**Health Behaviors**		
Physical Exercise	2009	66.1
Alcohol use	649	21.4
Smoking	557	18.3
** **	**TB**	**DLC**
**Age**	72.31	8.39
**Risk falling score on the CDC risk falling scale** (unit: score)	4.457	4.119

Table 2 shows that the mean score of knowledge regarding risk factors of falls was 11.37 ± 7.25 points. The highest scores were observed in terms of drug aspects, followed by medical condition and behavioural aspects.

**Table 2:** Knowledge regarding risk factors of falls among older people (n = 3038).

Knowledge (FRAQ)	Mean ± SD
Medical condition	3.75 **±** 2.58
Behaviours	2.13 **±** 1.46
Environment	1.15 ± 0. 87
Drug use	4.35 **±** 3.34
**Total**	**11.37 ± 7.25**

Table 3 shows that older people mostly agreed with items of perceived severity (mean = 15.28 ± 2.55), perceived susceptibility (mean = 18.23±2.38), perceived benefits (mean = 11.54 ±1.54) and perceived barriers (mean = 18.49±1.95) components. Meanwhile, the participants’ attitudes about cues to action (mean = 10.86±1.42), health motivation (mean = 6.52±1.17) and actions (mean = 12.97±2.33) components were most neutral.

**Table 3:** Attitudes of older adults about the severity of falls (n = 3038).

Contents	Totally disagree	Disagree	Neutral	Agree	Totally agree	Mean± SD
	%	%	%	%	%	
**Perceived Severity (4–20)**						**15.28±2.55**
Falls in the elderly are a very serious problem	0.7	0.6	30.5	55.0	13.2	3.79± 0.70
Falls in the elderly can cause fractures, disability and even death	0.5	0.8	28.7	56.0	14.0	3.82± 0.69
Falling in the elderly can alter the psyche and cause fear of falling.	0.7	0.7	27.6	57.0	14.0	3.83 ±0.69
Falls in the elderly can increase the burden on families.	0.1	0.5	29.2	56.2	14.0	3.83 ±0.66
**Perceived Susceptibility (5–25)**						**18.23±2.38**
Elderly people are prone to falls.	1.4	6.5	44.7	43.8	3.6	3.42 ±0.73
Lack of safety indoors and out of the community can lead to falls, such as slippery floors, tripping debris on walkways, etc.	1.4	6.5	47.2	41.5	3.4	3.39 ±0.72
Some bad habits can cause falls, including wearing inappropriate clothes and shoes, not using rails, etc.	0.8	1.8	26.1	60.8	10.4	3.78 ±0.68
Bad mental states can cause falls, such as depression	0.6	1.8	23.9	61.4	12.4	3.83± 0.68
Chronic diseases and impaired functioning of organs can cause falls.	0.5	0.9	27.3	59.9	11.5	3.81±0.66
**Perceived Benefits (3–15)**						**11.54 ±1.54**
Falls in the elderly can be prevented by appropriate methods	0.3	0.6	24.9	63.3	10.9	3.84±0.62
It is possible to reduce the risk of falling if I correct the lack of safety in the indoor environment.	0.6	0.5	27.6	57.3	14.0	3.83 ±0.68
Will reduce the risk of falling if I can change my bad habits	0.5	0.3	24.1	62.0	13.0	3.87±0.64
**Perceived Barriers (5–25)**						**18.49±1.95**
I know some bad habits but it’s hard for me to fix.	0.3	12.8	26.0	60.3	0.6	3.48±0.73
It’s hard for me to change some of the insecurity issues in my home environment.	0.8	1.5	27.4	61.5	8.8	3.76±0.66
It’s hard for me to determine the risk factor for falling.	0.7	1.1	34.2	54.0	10.0	3.72±0.68
It is very difficult for me to adhere to the treatment of chronic diseases that affect the fall, such as hypertension.	0.5	1.1	36.0	51.0	11.4	3.72±0.70
Prevention of falls is costly, such as installing handrails.	0.9	0.4	25.1	63.6	10.0	3.82±0.64
**Cues to Action (3–15)**						**10.86±1.42**
Fall prevention information on television, propaganda publications have an impact on me.	0.9	0.7	22.7	62.9	12.9	3.86±0.66
Fall experiences from family members and friends influenced me.	0.7	1.5	33.1	53.9	10.8	3.73±0.70
The views of family members and friends about the risks of falling and how to prevent them influenced me.	0.8	5.2	63.2	27.5	3.3	3.27±0.65
**Health motivation (2–10)**						**6.52±1.17**
I usually value my safety.	1.1	3.0	67.2	24.8	3.9	3.27±0.64
I often actively acquire knowledge of injury prevention	0.6	5.4	65.6	25.7	2.8	3.25±0.62
**Action (4–20)**						**12.97**±**2.33**
I am willing to participate in fall prevention activities.	1.1	3.1	67.5	24.6	3.8	3.23±0.64
I can complete the assigned tasks when participating in fall prevention activities.	1.1	3.1	67.5	24.6	3.8	3.27±0.63
I can resolve to correct my bad habits while participating in fall prevention activities.	0.9	5.4	65.5	25.5	2.8	3.24±0.63
I can change the insecurity issues in the home environment when participating in fall prevention activities.	0.9	5.0	67.0	24.6	2.5	3.32±0.62

Table 4 showed that more than half of the participants practiced recommended fall preventive measures. The mean score of fall prevention practices was 7.13/14.

**Table 4:** General fall prevention practices of older adults (n = 3038).

Practices	Yes		No	
	N	%	N	%
Remove obstacles such as papers, books, clothing, and footwear from stairs and frequently traveled places	2059	67.8	979	32.2
Use non-slip mats, or fix the carpets so as not to slip, do not spread loose mats in the house	1851	60.9	1187	39.1
Installing lighting and improving indoor lighting	1993	65.6	1045	34.4
Wear matching shoes with good support inside and outside the home	1718	56.6	1320	43.4
Install railings in stairs, bathrooms, toilets.	1547	50.9	1491	49.1
Keep frequently used items in your closet so they can be easily accessed without using stools or climbing up to retrieve items	1780	58.6	1258	41.4
Learn how to use assistive devices	1175	38.7	1863	61.3
Exercise regularly	1203	39.6	1835	60.4
Proper, healthy nutrition	1833	60.3	1205	39.7
Positive lifestyle changes such as reducing alcohol consumption, increasing daily exercise, etc.	1184	39.0	1854	61.0
Find out the risk of falling	1122	36.9	1916	63.1
Talk to family members, friends, or health providers about health conditions, drug use, vision. . . of himself	1829	60.2	1209	39.8
Regular health check-ups	1427	47.0	1611	53.0
Learn about fall prevention measures	950	31.3	2088	68.7
**Practice score**	**7.13 ± 4.48**			

Table 5 indicated that older people with history of fall in the last 12 months had significantly lower knowledge (Coef. = -8.459, 95%CI = -9.156; -7.762), attitude (Coef. = -1.581; 95%CI = -2.563; -0.599) and practice (Coef. = -5.923; 95%CI = -6.292; -5.554) scores compared to those without history of falls. In addition, compared to those living in 3-generation family, older people living in two-generation families had significantly lower attitude score (Coef. = -0.980; 95%CI = -1.612;-0.348).

**Table 5:** Factors related to knowledge scores, attitudes, and fall prevention practices.

Characteristics	Knowledge Score			Attitude Score			Practice Score		
	Coef.	95% CI		Coef.	95% CI		Coef.	95% CI	
**Age group** (vs. 60 to 69 years old)									
From 70 to 79 years old	0.221	-0.251	0.693	-0.429	-1.037	0.179	0.011	-0.252	0.275
Be 80 years of age or older	0.585	-0.017	1.186	-0.969*	-1.723	-0.216	-0.072	-0.406	0.262
**Occupation** (Retired vs Employed/Self-employed)	0.303	-0.131	0.737						
**Marital status** (vs. Single)									
Have a spouse	-0.959	-2.178	0.260				-0.449	-1.131	0.233
Separation/divorce	0.305	-1.978	2.588				-0.374	-1.651	0.903
Widow	-1.287	-2.610	0.036				-0.550	-1.290	0.190
**Family classification** (compared to 3-generation families)									
2-generations				-0.980**	-1.612	-0.348			
1-generation				-0.361	-1.078	0.356			
Living alone				-0.567	-2.273	1.139			
Others				-3.756	-9.321	1.808			
**History of fall** (None vs. Yes)	-8.459***	-9.156	-7.762	-1.581***	-2.563	-0.599	-5.923***	-6.292	-5.554
**Fall Risk Assessment** (No Risk of Fall vs. At Risk)	-0.670*	-1.215	-0.124	-0.617	-1.323	0.090	-0.351*	-0.656	-0.046
**Knowledge score**				-0.006	-0.052	0.040	0.016	-0.004	0.036
**Attitude score**	-0.002	-0.029	0.026				0.037***	0.021	0.052
**Practice score**	0.053	-.011	0.117	0.198***	0.116	0.280			

p<0.01

**p<0.05

*p<0.1

## Discussion

Our study partly contributed to the current body of literature about the knowledge, attitudes and practices of older people regarding risk factors of falls as well as fall prevention. Overall, older people in rural communes were not fully aware of their risk regarding falls even though they might have moderate levels of attitude and practices about fall prevention.

In this study, we examined four types of fall risk factors including medical conditions, behaviours, environment and drug use. The results of this study showed a low level of knowledge among our participants, which was much lower than other previous studies [20–23]. A study conducted in the United States explored the prioritization of competing health risks among older adults, revealing that a mere fraction of less than 10% regarded the prevention of fall injuries as their utmost concern [24]. Other studies have highlighted the fact that older individuals possess a comprehension of the significance of factors associated with the risk of falling [25, 26]. However, they fail to acknowledge their susceptibility to these risks [27]. This perceived absence of risk for falls serves as a significant obstacle to seniors engaging in fall prevention initiatives [28]. Consequently, if older individuals do not acknowledge their vulnerability to falls, the likelihood of them seeking guidance from medical professionals on ways to mitigate such risks may be diminished. In the present study, the underlying causes for the limited knowledge observed can be attributed to the lack of comprehensive health education programs focusing on fall prevention within Vietnam, particularly in rural regions, where older individuals are disproportionately affected. Moreover, the older population exhibits subjectivity regarding fall prevention and lacks awareness of their susceptibility to falls.

Regarding attitude towards fall prevention, according to the Health Belief Model (HBM), people who perceived the severity of health problems were more likely to engage in behaviours to prevent these problems from occurring (or reduce their severity) [29]. Our finding was consistent with a previous study which indicated that older people mostly perceived the severity of falls [30]. This aspect could potentially provide an advantage, as individuals may be more inclined to engage in preventative measures aimed at mitigating the risk of falls and recurrent falls. The effectiveness of implementing these measures depends upon their comprehension and awareness of the preventative measures in question.

Similarly, when assessing the perception of older people’s susceptibility to a specific health issue would be inclined to adopt behaviours aimed at mitigating their risk of experiencing said health problem. Individuals demonstrating a diminished level of perceived susceptibility tend to exhibit an inclination to dismiss their vulnerability to health complications, consequently leading them to partake in behaviours that are detrimental to their well-being or carry inherent risks. In contrast, individuals who held a heightened perception of susceptibility to a specific health issue were inclined to actively engage in mitigating behaviours to decrease their chances of acquiring the said condition [29]. Our study’s findings indicate that older adults largely demonstrated a positive disposition towards susceptibility, surpassing that observed in previous research [30]. According to the findings, it can be inferred that older adults possess a perception of being highly vulnerable to risks and manifest a willingness to engage in interventions aiming to modify behaviours and ultimately diminish said risks.

However, when evaluating perceived barriers regarding the modification of risk factors for falls, it was observed that a majority of older adults expressed difficulty in changing aspects related to their behaviours or daily living environments, such as the challenging task of modifying the structure of their homes or unsafe behaviours. According to the HBM, these barriers pose significant obstacles to the ability to modify behaviours and engage in healthcare activities [29], particularly in the case of fall prevention. The following are the issues that healthcare planners and professionals need to consider and incorporate into their plans when implementing interventions for preventing falls in older adults.

This study also examined other aspects such as cues to action, health motivation, and action that can encourage older people to participate in health-protective behaviours and fall prevention. Older people in this present study showed a moderate attitude towards these aspects, which was in line with prior research [30]. Generally, cues that facilitate the initiation of action may encompass televised fall prevention information, health education publications, shared anecdotes of fall experiences from family and acquaintances, the perspectives of loved ones regarding the hazards associated with falls and preventive measures that impact the older population, as well as individual motivation manifested in the recognition of personal safety, the active pursuit of injury prevention knowledge, and the unwavering resolve to effect change. Nevertheless, in Vietnam, a community-based fall prevention intervention program dedicated to the elderly population was notably rare. Furthermore, variations in individuals’ levels of self-motivation were observed. When individuals endeavour to adopt health-protective behaviours, they encounter challenges in addressing preexisting detrimental habits and overcoming obstacles and complexities associated with preventive actions. To exhibit optimal and appropriate conduct, the older population must possess a comprehensive and thorough understanding of strategies for mitigating falls.

The level of engagement of older adults with various fall prevention strategies was found to be relatively low from 30% to 60%, especially several preventive measures such as using assistive devices, exercising regularly; changing a positive lifestyle such as reducing alcohol consumption, and increasing daily exercise. Even with other measures that supported them in raising awareness of the risks of falling off, and learning about fall prevention measures, their participation was low. However, according to the regression analysis, older individuals who had a higher level of attitude toward fall prevention were significantly more inclined to engage in prevention activities, which aligned with previous research [7]. The aforementioned point holds critical significance, as it posits that augmenting one’s comprehension regarding the significance of fall prevention for both health and safety can potentially enhance the adoption of fall prevention measures. The messaging aimed towards older people regarding fall prevention should prioritize emphasizing the positive impacts on health and social well-being, while also portraying it as a means of enhancing their overall quality of life.

This study had several implications. The outcomes of this study indicate the necessity for healthcare practitioners to provide comprehensive and articulate instructions regarding medication administration, potential adverse effects, and the identification of abnormal signs resulting from diverse pharmacological interventions. Given the lack of fall prevention interventions and rehabilitation centers in Vietnamese rural communities, particular emphasis should be placed on instructing older people on how to accurately record pertinent details such as medication names, dosages, and the frequency of daily usage. Moreover, the findings indicate that local healthcare workers should plan and perform health education interventions, as well as implement health services related to fall prevention in the rural community such as strength and balance training, as well as other practices that the older people were difficult to perform at home. Particularly, older people in rural community often participated in agriculture jobs such as farmers, it is suggested that the development of targeted fall prevention messaging directed towards older adults is necessary, especially guidelines for those who active participated in farming works. Efforts aimed at reaching the elderly demographic ought to consider communicating the significance of fall prevention in promoting the overall health of older adults. These messages should adopt an informative approach that educates this population about specific practices focused on preventing falls. Additionally, it is crucial to underscore the importance and efficacy of fall prevention strategies in preserving the functionality, independence, and well-being of older adults.

The strength of this study included the large sample size and the use of different instruments to measure risk as well as the knowledge, attitude and practice of older people regarding fall prevention. However, several limitations should be noted. First, the study employed a cross-sectional design, precluding the ability to establish a causal relationship between knowledge, attitude and practice with the potential associated factors, requiring additional longitudinal research to fill this evidence gap. Second, despite a large sample size, our study was performed in some locals which might not be representative of other locations that had different demographic, cultural and health service characteristics. Third, environmental factors such as house characteristics, social relationships or exposure to any healthcare programs were not assessed in our study, which might play an important role in forming knowledge, attitudes and practices of older people. Furthermore, our support for the instrument’s validity is constrained to face and content validity, as well as factor analysis. The robustness of the evidence could have been enhanced with the inclusion of additional objective measures of validity. Moreover, our survey, which employed closed-ended questions, only managed to capture certain dimensions of knowledge, attitude and practice. It is imperative to conduct additional research to substantiate the levels of knowledge and practice by using open-ended questions or conducting direct observations. This study did not assess individuals’ knowledge regarding fall prevention practices nor did it evaluate their active engagement in such practices. However, it is plausible to consider that an increased understanding of the negative consequences of falls and the implementation of preventive measures could potentially impact individuals’ priorities.

## Conclusion

This study showed limited knowledge regarding risk factors of falls, as well as a moderate level of attitude and practices on fall prevention among older adults. Health education interventions and fall prevention services that enhance community-based fall prevention knowledge, attitudes and practices for older adults should be performed to reduce the burden of falls in this population.
